# Unlocking the biosynthetic potential and taxonomy of the Antarctic microbiome along temporal and spatial gradients

**DOI:** 10.1128/spectrum.00244-24

**Published:** 2024-05-15

**Authors:** William Medeiros, Kelly Hidalgo, Tiago Leão, Lucas Miguel de Carvalho, Nadine Ziemert, Valeria Oliveira

**Affiliations:** 1 Microbial Resources Division, Research Center for Chemistry, Biology, and Agriculture (CPQBA), Universidade Estadual de Campinas (UNICAMP), Paulínia, São Paulo, Brazil; 2 Interfaculty Institute of Microbiology, and Infection Medicine Institute for Bioinformatics and Medical Informatics, German Centre for Infection Research (DZIF), Tübingen, Germany; 3 Chemistry Institute, São Paulo State University (UNESP), Araraquara, São Paulo, Brazil; 4 Center for Computing in Engineering and Sciences, Universidade Estadual de Campinas (UNICAMP), Campinas, São Paulo, Brazil; University of Porto, Porto, Portugal

**Keywords:** biosynthetic gene clusters, metagenome mining, Antarctic environments, Whalers Bay

## Abstract

Extreme environments, such as Antarctica, select microbial communities that display a range of evolutionary strategies to survive and thrive under harsh environmental conditions. These include a diversity of specialized metabolites, which have the potential to be a source for new natural product discovery. Efforts using (meta)genome mining approaches to identify and understand biosynthetic gene clusters in Antarctica are still scarce, and the extent of their diversity and distribution patterns in the environment have yet to be discovered. Herein, we investigated the biosynthetic gene diversity of the biofilm microbial community of Whalers Bay, Deception Island, in the Antarctic Peninsula and revealed its distribution patterns along spatial and temporal gradients by applying metagenome mining approaches and multivariable analysis. The results showed that the Whalers Bay microbial community harbors a great diversity of biosynthetic gene clusters distributed into seven classes, with terpene being the most abundant. The phyla Proteobacteria and Bacteroidota were the most abundant in the microbial community and contributed significantly to the biosynthetic gene abundances in Whalers Bay. Furthermore, the results highlighted a significant correlation between the distribution of biosynthetic genes and taxonomic diversity, emphasizing the intricate interplay between microbial taxonomy and their potential for specialized metabolite production.

**IMPORTANCE:**

This research on antarctic microbial biosynthetic diversity in Whalers Bay, Deception Island, unveils the hidden potential of extreme environments for natural product discovery. By employing metagenomic techniques, the research highlights the extensive diversity of biosynthetic gene clusters and identifies key microbial phyla, Proteobacteria and Bacteroidota, as significant contributors. The correlation between taxonomic diversity and biosynthetic gene distribution underscores the intricate interplay governing specialized metabolite production. These findings are crucial for understanding microbial adaptation in extreme environments and hold significant implications for bioprospecting initiatives. The study opens avenues for discovering novel bioactive compounds with potential applications in medicine and industry, emphasizing the importance of preserving and exploring these polyextreme ecosystems to advance biotechnological and pharmaceutical research.

## INTRODUCTION

Soil microbial communities are responsible for synthesizing a diverse set of specialized metabolites that represent a rich source for drug discovery. Most of these molecules are produced as part of secondary metabolism and are critical chemical compounds for microorganisms to interact with the environment. They play vital ecological roles in the complex network of microbial communities, including communication, nutrient uptake, and defense against predation (1). Beyond their ecological functions, these bacterial molecules are widely applied in industry, agriculture, and medicine as natural products, such as microbial pigments, antibiotics, immunosuppressants, and anti-cancer drugs (2, 3). These specialized metabolites are encoded by biosynthetic gene clusters (BGCs), groups of physically clustered genes that code for the enzymes necessary to synthesize the specialized metabolite (4, 5). The structural arrangement of BGCs facilitates their identification, and a great diversity of BGCs have been identified using several tools that were developed for mining genomes for these gene clusters, including AntiSMASH (6), PRISM (7), and DeepBGC (8). However, despite the growing efforts that have been made to understand BGC functions in nature, little is known about how environmental variables drive their distribution in soil, and even the extent of their diversity is not well understood (9–11).

Likewise, studies about the diversity of BGCs and the biotechnological potential of encoded compounds in the Antarctic microbiome are at an early stage. Microorganisms that inhabit extreme environments need to develop biological adaptations, mechanisms, and strategies in response to harsh environmental conditions, such as extreme temperatures, desiccation, high values of UV radiation, and pH (12). Thus, microbial communities adapted to extreme environments may represent reservoirs of metabolic products. Some studies on this topic can be highlighted, including the one conducted by Liao et al. (13), who used genome mining to investigate the genomic potential of *Marisediminicola antarctica*, a species endemic to Antarctica from the bacterial order Actinomycetales, and one conducted by Rego et al. (14), who investigated BGC classes of non-ribosomal peptide synthetase (NRPS) and polyketide synthase (PKS) and demonstrated the new chemical diversity in Maxwell Bay, Antarctica.

However, approaches based on metagenomics that allow the identification of BGCs of interest and their distribution across environments are still scarce in the literature. In addition, multiple factors can influence the distribution of BGCs in the environment. Even ecologically similar yet geographically distant environments may harbor distinct groups of bacterial species, resulting in differences in the composition of BGCs (15). Furthermore, environmental variables such as latitude and pH have been observed to correlate with alterations in the structure of biosynthetic domains, implying that both soil-related and non-soil-related factors contribute to determining the BGC content of microbiomes (16). It is also likely that a relatively small global collection of largely conserved BGCs occurs across different locations (5). Further investigation is required to comprehensively understand the factors influencing the distribution of BGCs in the environment. At the same time, Antarctica represents a vast reservoir of microorganisms with biosynthetic potential for new natural products and one of the least known environments (13, 17).

Herein, we investigated the BGC diversity of the microbial community in sediments of Whalers Bay, in Deception Island, Maritime Antarctica. Deception is an active volcano on the Antarctic Peninsula. A previous metagenomic study focused on characterizing the microbial resistome profile showed that *Psychrobacter* and *Polaromonas* (Proteobacteria) are the main genera harboring resistance/tolerance genes and that the resistome profile significantly varied along the Whalers Bay transect line (18). However, natural product biosynthetic gene diversity in the Whalers Bay microbial community is still unknown. The volcanic nature of Deception Island soil and the geographic isolation plus the Antarctic extreme environmental conditions provide a particular and polyextreme environment that has likely selected biosynthetic genes encoding novel and unique bioactive functions (17). Thus, this study aimed to evaluate the BGC diversity and novelty and to assess their differential abundance distribution along a spatiotemporal gradient.

## MATERIALS AND METHODS

### Metagenomic data sets and metadata

Sampling procedures, metadata assessment, DNA extraction, and sequencing were previously performed and described by Centurion et al. (19). Briefly, the research team of the MycoAntar Project, supported by the Brazilian Antarctic Program, PROANTAR, collected biofilm sediment samples, in replicates, in Whalers Bay along a transect line during the summers of 2014, 2015, and 2017. The transect line encompassed four sampling sites, ranging up to 33 m from near the glacier toward the coast in 2014 and 2017, and three sample sites, ranging up to 20 m in 2015 (Fig. S1). Physicochemical parameters of each sample were assessed (Table S1), and the DNA extraction and shotgun sequencing were conducted using PowerSoil kit (Qiagen, Inc., Hilden, Germany) and the Illumina HiSeq (2 × 150, paired end) platform, respectively. The sequence data generated can be accessed under the project number PRJEB29861 (http://www.ebi.ac.uk).

### Bioinformatic analyses

Sequence quality was evaluated using FastQC software v.0.11.9 (20), and low-quality sequences (Phred score <30) were trimmed using the Trimmomatic tool v.036 (21). Then, the average coverage of the metagenomic data sets was estimated using Nonpareil v.3.4 (22). Sequences were further assembled into contigs using the megahit tool (23) in the co-assembly mode, and the assembly quality was evaluated using Quast v.5.0.2 tool (24). Finally, the taxonomic profile was assessed using the Kraken2 v.2.1.2 (25) algorithm with GTDB database release 207.2 (26). Bowtie2 v.2.3.5.1 (27) was used for mapping the reads to the metagenomes recovered (contigs) and for calculating the relative abundance of the taxonomic groups.

#### Metagenome mining

The metagenomes were mined for BGCs using AntiSMASH v.5 (6) in a local server with the following functions enabled: --cb-knownclusters --cb-subclusters –genefinding-tool prodigal –asf –pfam2go –smcog-trees –cc-mibig –rre –fullhmmer. Furthermore, BGCs were clustered into gene cluster families (GCFs) using a similarity threshold of 0.3 by calculating a (dis)similarity matrix using the BiG-SCAPE algorithm (28). The GCFs were formed based on a combination of three metrics: Jaccard index, adjacency index, and conserved domain sequence similarity. Accordingly, it was possible to evaluate the GCFs formed for each class of BGC as a unique unit, similar to the operational taxonomic unit used for metataxonomic analysis, and then investigate the variation from the genetic composition content across the samples. The BiG-MAP software (29), consisting of Python modules, was used as a normalization method to calculate gene abundances in the metagenome data set and to evaluate the differential abundances among samples. Finally, Nerpa (30) was applied to link BGCs of NRPSs with their respective specialized metabolites through a computational method that combines data from metagenome mining with known chemical structures of non-ribosomal peptides (NRPs).

### Statistical analyses

The tables with BGCs, GCFs, taxonomy, and metadata annotations were exported to the R Statistical environment. Tidyverse packages (31) were used, including several other packages, for data analyses. For multivariate analysis and visualization, Factorextra (32), Factorminer (33), and Vegan (34) were used. Analysis of variance (ANOVA) was performed to analyze the differences among BGCs’ relative abundances with the post hoc Tukey test (*P* value ≤0.05). The correlation between BGCs’ relative abundances and environmental data was obtained using canonical correspondence analysis (CCA). For data visualization, ggplot2 package (35), cytoscape (36), and RAWGraphs (37) were applied.

## RESULTS AND DISCUSSION

### BGC diversity in the Whalers Bay biofilm microbial community

**Fig 1:**
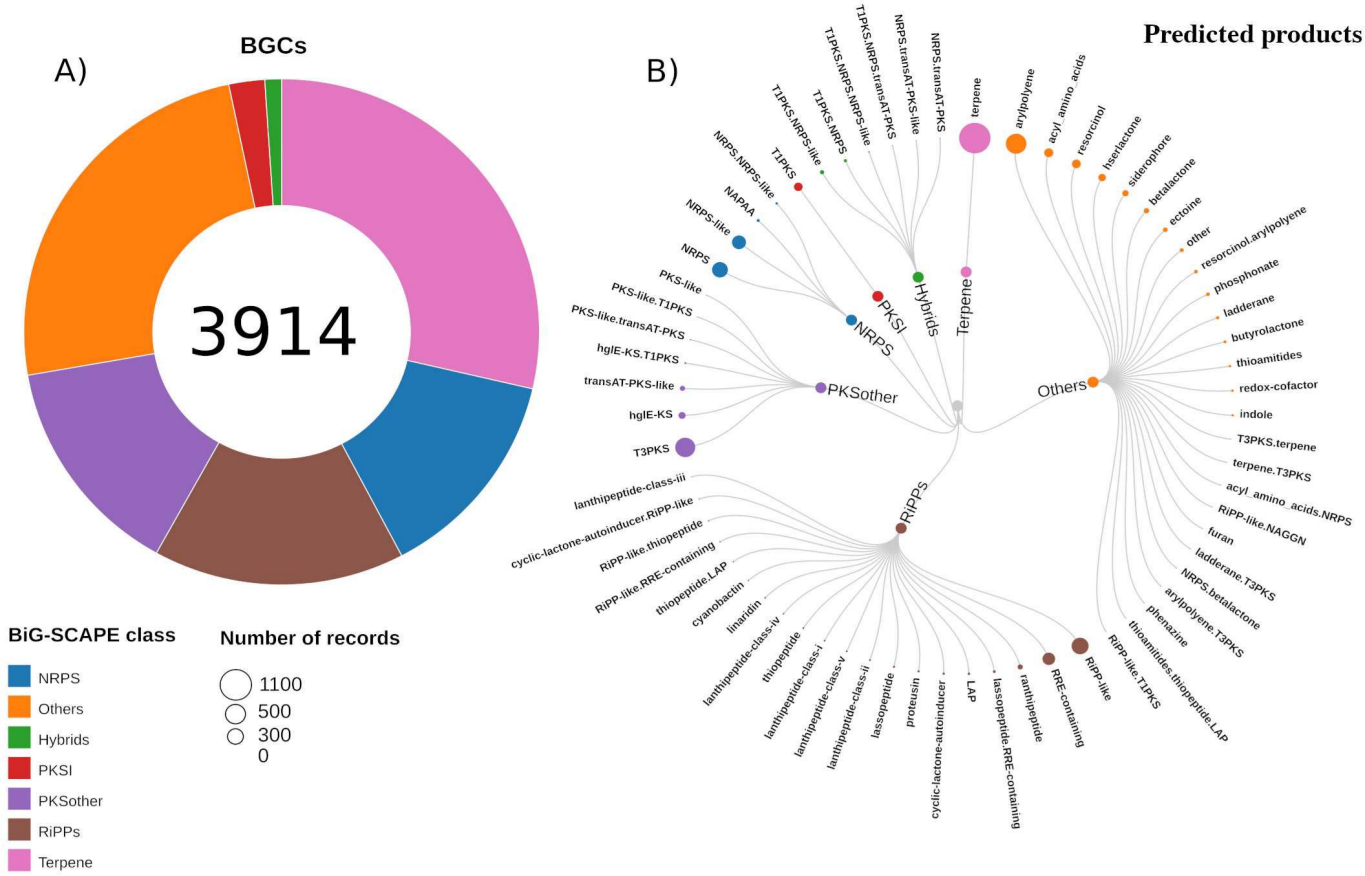
Biosynthetic gene cluster (BGC) classes and predicted products identified in the Whalers Bay biofilm microbial community. (**A**) Abundances of BGC classes. (**B**) Predicted products encoded within each BGC class. Circle sizes correspond to the predicted product abundances.

To comprehensively assess the BGC diversity within the Whalers Bay biofilm microbial community, all samples were analyzed together. Around 3.7 million contigs were assembled, of which 610,924, 1,130,529, and 2,032,259 were recovered from 2014, 2015, and 2017 samples, respectively (Table S2). Although the data sets showed differences in sequencing effort, coverage estimate was above 60% for all data sets (Fig. S2), which represents sufficient coverage to ensure assembly quality and detection of differential gene abundance (38). A total of 3,914 BGCs were identified and classified into seven different BGC classes according to AntiSMASH v.5.0 classification (Fig. 1A). Out of the total BGCs recovered, only 75 were not identified as located at the contig edge by AntiSMASH. This observation suggests that the majority of the recovered BGCs may not be complete. Notably, mining metagenomic data sets for BGCs often yields BGCs located at the contig edge. For example, Waschulin et al. (39) demonstrated that 96.7% of recovered BGCs from short-read sequencing technology were annotated as being on a contig edge. Nevertheless, the recovered BGCs ranged in average length from 4,326 to 19,960 bp (Table 1), enabling the exploration of secondary metabolic potential within microbial genomes and the identification of similarities to known products. Terpene appeared as the most abundant BGC class, which represented around 30% of total BGCs identified. These findings are corroborated by the study of Waschulin et al. (39), who observed terpene as the most abundant BGC class in Mar Oasis, Southeast of Alexander Island, in the Maritime Antarctica. Similar results were obtained by Benaud et al. (40), which showed terpenes as the most abundant BGCs found in Antarctic bacterial isolates, and by Rego et al. (41) in a metagenomic study of the Arctic Ocean. Terpene synthase genes are widely distributed in bacteria (42) and stand out as one of the most diverse natural product classes. These compounds exhibit high chemical structure diversity and ecological roles (43), such as modulation of membrane fluidity and protection against cold stress, maintaining homeostasis regarding membrane fluidity in response to temperature fluctuations in psychrophilic bacteria (44). Therefore, the great terpene abundance was expected based on the ecological role that this compound class plays regarding membrane stability in a cold-stressed environment (45–47) and the protection against UV radiation (1, 48).

**TABLE 1:** Summary of biosynthetic gene clusters grouped into gene cluster families^*a*^

	PKSI^*b*^	PKSother	PKS-NRP_hybrids	NRPS	RiPPs	Terpene	Other
No. of BGCs	89	602	41	578	621	1,123	860
Average BGC length (bp)	4,326	5,782	19,960	5,094	7,000	6,258	6,602
No. of families	79	362	33	497	444	727	597
Average no. of BGCs per family	1	2	1	1	1	2	2
Maximum no. of BGCs in a family	3	39	4	5	12	15	16
Singletons	71	281	27	431	341	559	456
Percentage of singletons (%)	89.9	77.6	81.8	86.7	76.8	76.9	76.4
Families with MIBiG reference BGCs	0	2	0	0	0	1	1

^*a*^ Cutoff ≥70% similarity was applied for BGC clustering.

^*b*^ PKSI, polyketide synthase type I; RiPP, ribosomally synthesized and post-translationally modified peptide.

Metagenome mining also revealed that the biofilm microbiome sampled from the Whalers Bay sediments harbors a vast number of BGC classes of interest in drug discovery since these BGCs encode enzymes that synthesize antibiotics and immunosuppressive molecules. A total of 602 (~15%) BGCs of PKSother (which includes PKS types II and III), 578 (~14%) of NRPS, and 89 (~2%) PKS type I were identified (Fig. 1A). Moreover, the AntiSMASH algorithm predicted a wide variety of products encoded in each BGC class (Fig. 1B). For instance, within the BGC class “others,” the predicted products aryl polyenes were found as the most abundant. Aryl polyenes are specialized polyunsaturated carboxylic acids widespread in bacteria. These molecules form yellow pigments widely distributed in different environments, playing vital ecological roles in intra- and interspecific interaction of the microbial community and in responses to environmental changes, for example, promoting the biofilm formation and exhibiting antioxidant protection, similar to the protection function observed in carotenoids (49, 50). Additionally, ribosomally synthesized and post-translationally modified peptides (RiPPs) were predicted to encode diverse product classes, such as cyanobactins, which include anti-malaric and anti-tumor compounds (51); lasso peptides, a group of peptides that exhibit anti-microbial activity and anti-cancer properties (52, 53); and thiopeptides, molecules that display antibiotic activity with distinct chemical structures and a wide range of biological activities (54).

Based on genetic distances calculated through the BiG-SCAPE algorithm (28), all BGCs identified were clustered into their respective GCFs (Table 1). Terpenes formed the most abundant GCF (727), followed by NRPS (497) and RiPPs (444). In general, all GCFs showed a high percentage of singletons (cutoff ≥70% similarity), which indicates a diverse genetic composition. Among the BGC classes, PKSI contained the most diverse GCFs, with around 90% of GCFs as singletons and the remaining containing a maximum of three BGCs per GCF (Table 1; Fig. S3). The significant number of singletons reflected the lower complexity of PKSI network connections compared to RiPPs, which showed the lowest genetic composition diversity (around 76% of singletons) (Table 1; Fig. S3). Besides that, the abundances of NRPs, PKSI, and PKSother taken together represented around 34% of the total GCFs identified in the Whalers Bay biofilm microbial community. These classes encode a broad diversity of bioactive compounds with pharmaceutical applicability, such as actinomycin, encoded by NRPS, and erythromycin, encoded by PKS (55). Interestingly, among 3,914 BGCs grouped into 2,739 GCFs, only 4 GCFs formed clusters with the MIBiG v.3.0 database (Fig. S3), a curated repository containing BGCs of characterized compounds (56), revealing a high degree of novelty among BGCs found in the Whalers Bay biofilm microbial community. From those clustering with MIBiG reference BGCs, two belonged to PKSother (BGC0000281.1 and BGC0000938.1) and exhibit anti-phytopathogenic properties and activity against multidrug-resistant Gram-negative bacteria, respectively (57, 58). The third one was a terpene (BGC0000647.1) described as carotenoid (59), and the fourth one was classified by the BiG-SCAPE software as others (BGC0000938.1), belonging to a specialized metabolite described as fosfomycin, a broad-spectrum antibiotic (58).

The exact number of BGCs must be carefully considered due to the constraints of short-read technology on recovering full-length clusters (60), which produce BGCs split into different contigs (39), leading to an overestimation of BGCs. Nonetheless, the findings described herein indicate that the biosynthetic genes identified in the Whalers Bay biofilm microbial community are primarily unknown and could likely encode specialized metabolites that might present a bioactive novelty, highlighting the untapped huge biosynthetic potential of the Antarctic microbes.

#### BGC diversity and distribution in bacterial taxonomic groups

**Fig 2:**
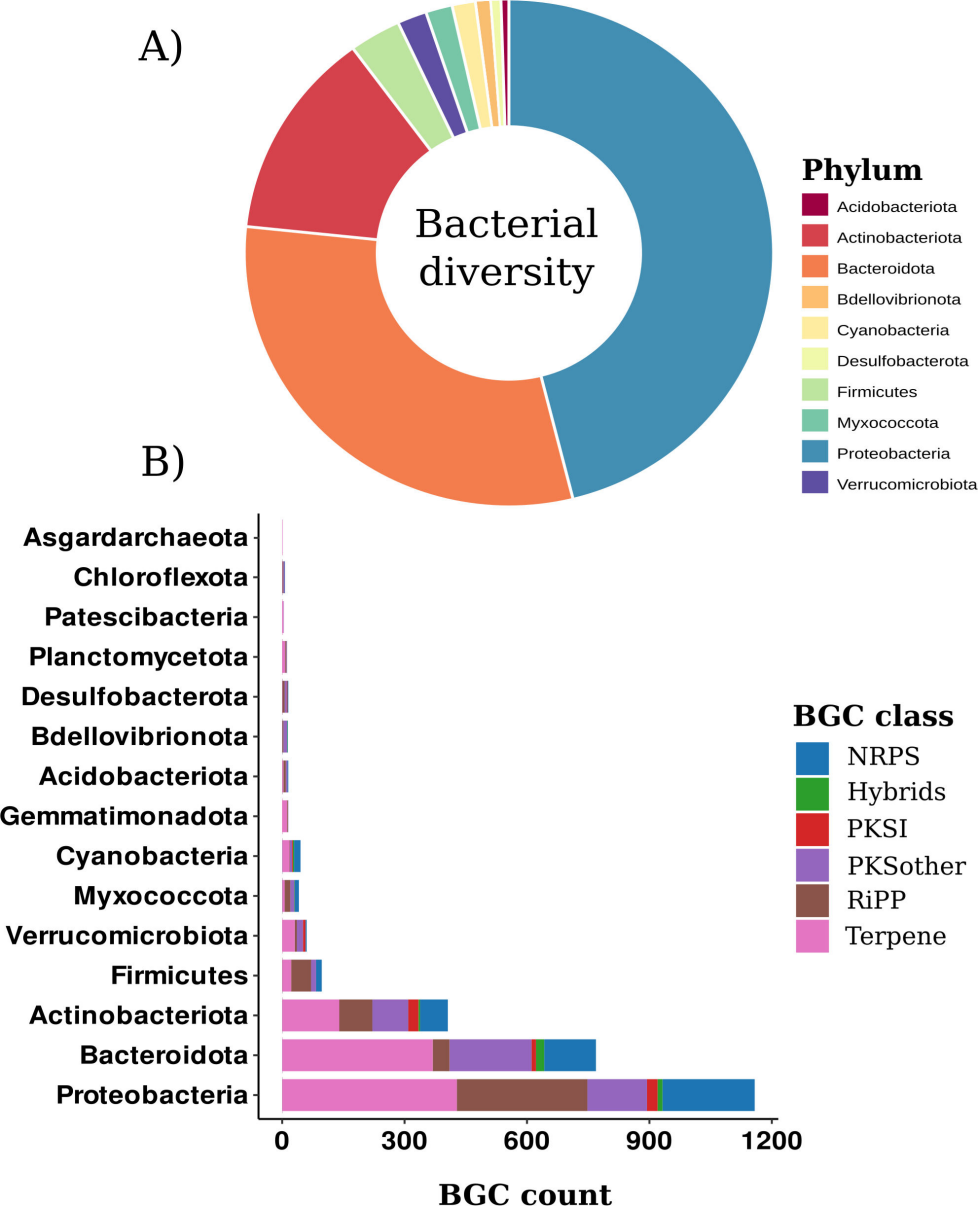
Bacterial diversity in the Whalers Bay Biofilm of Deception Island, Antarctica, and biosynthetic gene cluster (BGC) counts by phylum. (**A**) Relative abundances of the top 10 bacterial phyla identified in Whalers Bay. (**B**) Distribution and counts of BGCs within the top 15 phyla harboring distinct BGC classes.

The phylum Proteobacteria showed the highest relative abundance in the microbial community of Whalers Bay biofilm, followed by Bacteroidota and Actinobacteriota (Fig. 2A). These phyla were also the ones that mostly contributed to BGC counts in Whalers Bay (Fig. 2B). Similar patterns were observed in Mar Oasis, Maritime Antarctica (39), where Proteobacteria were contributing to the highest number of BGCs. However, while Actinobacteriota and Acidobacteriota also showed great BGC counts in Mar Oasis, Proteobacteria and Bacteroidota seemed to be significantly more associated with BGC abundance in Whalers Bay (Fig. 2B). In addition, in Whalers Bay, Acidobacteriota did not show relevance regarding BGC abundances, while in the desert soil of Antarctica, Actinobacteriota and Proteobacteria together accounted for more than 50% of the bacterial population and appeared as the most important producers of bioactive natural products (40). These results indicate that, although Proteobacteria, Bacteroidota, Actinobacteriota, and Acidobacteriota are ubiquitous in Antarctic microbial communities [see Doytchinov and Dimov (17) for a detailed review], site and local environmental conditions may select for specific abundances of the different phyla (61–63) and of biosynthetic genes encoding specialized metabolites (9, 64). Besides those phyla, Firmicutes, Verrucomicrobiota, and Cyanobacteria also showed high relative abundances (Fig. 2A) and harbored a great number of BGCs (Fig. 2B). Moreover, terpene, RiPP, NRPs, and PKS were present in the most abundant phyla. Terpenes are the BGC class more broadly distributed in all phyla, and NRPs showed stable relative abundance across the phyla Proteobacteria, Bacteroidota, Actinobacteriota, Firmicutes, Myxococcota, and Cyanobacteria.

#### Linking BGCs to non-ribosomal peptides with known function

**Fig 3:**
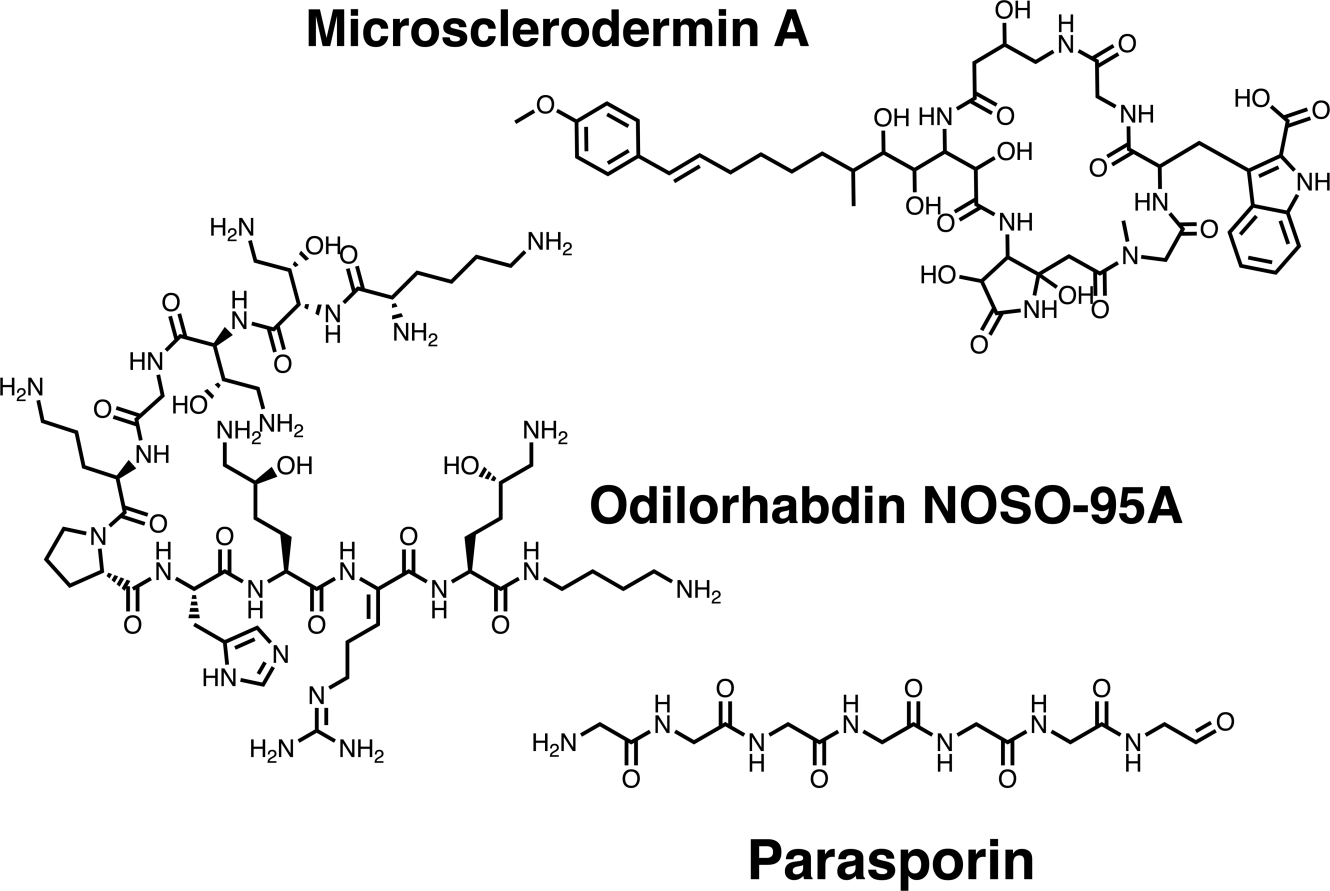
Chemical structures of known non-ribosomal peptides connected to BGCs identified in Whalers Bay, Deception Island, Antarctica.

Given the pivotal role of NRPs in synthesizing diverse specialized metabolites of clinical relevance, linking orphan NRPs BGCs to known compounds constitutes a strategic initiative aimed at discovering bioactive compounds and harnessing previously unexplored biosynthetic potential. To connect the BGCs of NRPs to the corresponding specialized metabolites, a computational approach that combines predicted chemical structures from genome mining data to known NRPs was applied (30). Using this approach, 44 NRPs were computationally linked to their corresponding encoded peptide (score ≥6), revealing a wide diversity of biological functions, such as anti-fungal, anti-cancer, and a new antibiotic class (Table S3). Among these linked BGCs, some putative specialized metabolites found stood out due their biological function (Fig. 3), including microsclerodermin, which exhibits a potent anti-fungal activity (65); parasporin, a protein with preferential cytotoxic activity for human cancer cells of diverse origins (66–68); and odilorhabdin NOSO-95A, described as a new class of antibiotic with activity against Gram-positive and Gram-negative bacteria through binding to the small subunit of the bacterial ribosome, causing mistranslation of proteins and adding premature stop codons (69).

Despite the persistent challenge of reliably linking BGCs to their corresponding specialized metabolites in natural product discovery, Kunyavskaya et al. (30) demonstrated that the Nerpa approach successfully predicted the production of ngercheumicin peptides by *Photobacterium galathea*, a prediction later confirmed through experimental validation (30). Nerpa provides a scoring system that assesses the likelihood of a predicted NRP sequence to align with the BGC module predictions, taking into account factors such as core amino acid specificity, methylation, stereochemistry, and potential insertions or deletions. The authors also established a recommended score cutoff of ≥6, which maintained a false-positive rate below 50% for amino acid matching. Here, from all BGCs of NRPs identified (578), only 44 matched to known NRPs (score ≥6), representing around 7% of the total NRPs found in the Whalers Bay biofilm microbial community. This result may help differentiate BGCs encoding known specialized metabolites from putative novel metabolites, which could further provide insights for prioritizing BGCs for NRP heterologous expression or for bioengineering novel variants of the known compounds that exhibit biological activities with medical interest. Additionally, the results demonstrated that Whalers Bay harbors a diverse biosynthetic arsenal encompassing biological functions and chemical structures yet to be discovered.

### BGC distribution across a temporal gradient in the Whalers Bay biofilm microbial community

The abundance of BGC-predicted products was assessed in the Whalers Bay biofilm across a temporal gradient spanning the summers of 2014, 2015, and 2017 (designated as WB14, WB15, and WB17, respectively). Samples were grouped according to the year of sampling, and statistical analysis was performed using ANOVA, followed by post hoc Tukey test to identify significant differences (*P* value ≤0.05). Aryl polyenes, heterocyst glycolipid synthase-like (*hgl*E), homoserine lactones (hserlactones), PKSI, RiPP, RiPP recognition element, siderophores, PKS type III, terpenes, and thioamides showed higher abundances in WB14 and remained statistically stable in WB15 and WB17 (Fig. S4). Only butyrolactone and trans-AT PKS (trans-AT) showed lower abundances in WB14 than the other predicted products and did not exhibit statistically significant differences between WB15 and WB17. In several *Streptomyces* spp., antibiotic biosynthesis is often coordinated by the signaling molecules gamma-butyrolactones (70, 71), and trans-ATs have been mainly described from non-culturable bacteria, belonging to the polyketide synthetase classes. Trans-ATs are involved in the biosynthesis of a wide variety of bioactive polyketides, representing a great potential for drug discovery (72). The temporal variations observed in BGC-predicted product abundances highlight the need to perform sampling at different times for the same site to properly identify hotspot regions harboring biosynthetic genes of interest. The other predicted products, such as resorcinol, acyl amino acids, lasso peptides, and NRPS, did not show significant differences between the years, probably due to the crucial ecological roles of these specialized metabolites in the environment (1).

The PKSI class showed higher abundances in WB14 (Fig. S4), and NRPS did not show statistical differences between the sampling years. Moreover, both BGC classes demonstrated high abundances in every year sampled. The PKSI and NRPS BGC classes encode a remarkable diversity of chemical structures and are pivotal in the biosynthesis of a broad spectrum of specialized metabolites with clinical relevance. Consequently, illuminating their diversity and distribution in extreme environments is of utmost importance for comprehending their roles in microbial ecology under the limits of life and exploring their potential in biotechnology. Thus, to deepen our understanding of PKSI and NRPS diversity and distribution, their genetic diversity along the years was evaluated using the BGCs grouped into GCFs.

**Fig 4:**
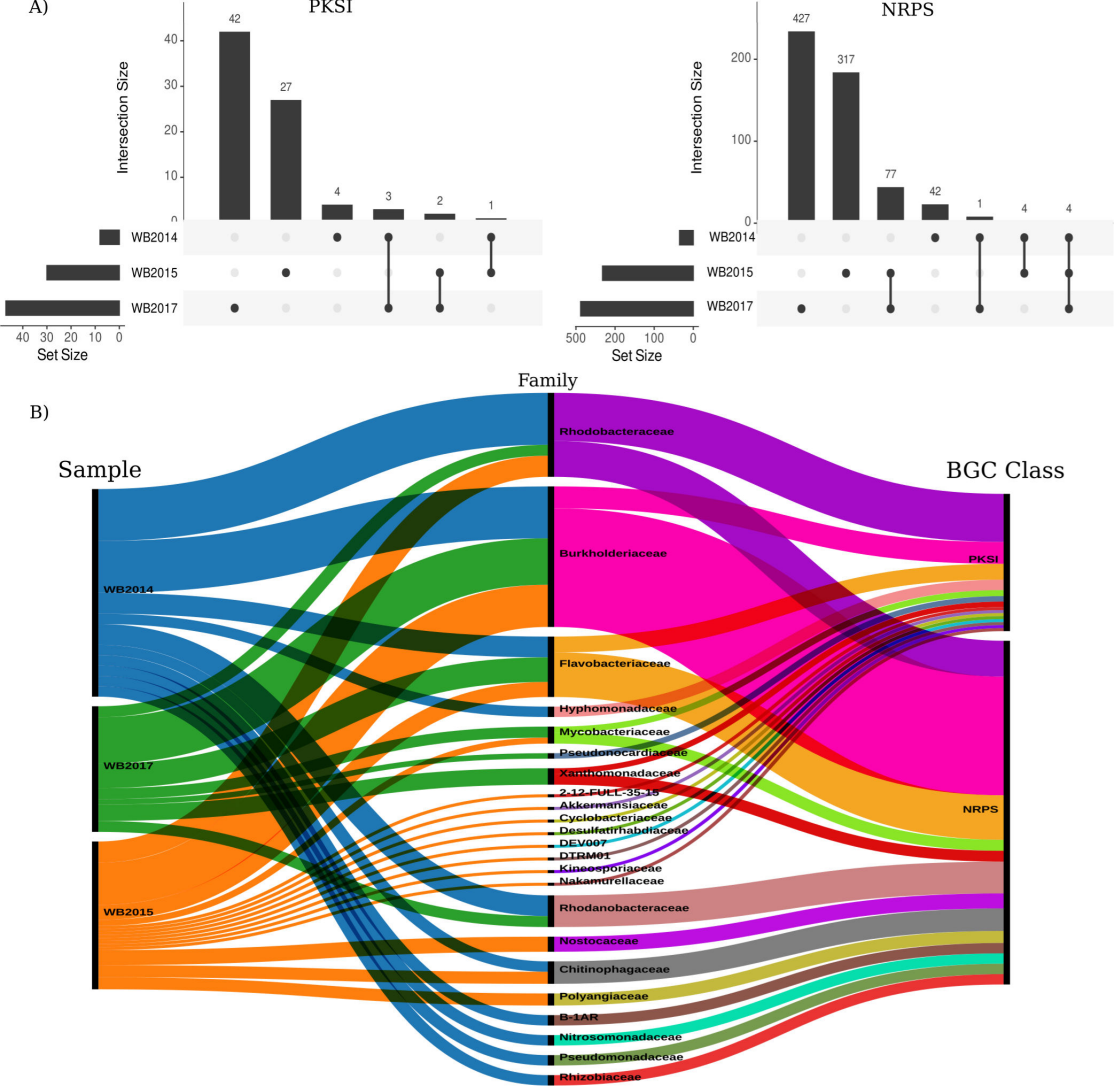
PKSI and NRPS intersections and distribution. (**A**) The upset plot illustrates the overlap of PKSI and NRPS clusters observed over the sampled years. The top bar plot in each panel indicates the intersection size (number of clusters) in the combined sample sets, as reflected in the matrix below. Shared clusters among samples are denoted by dots connected with straight lines. (**B**) Taxonomic family distribution within samples harboring PKSI and NRPS clusters.

Interestingly, PKSI and NRPS exhibited high replacement rates across the temporal gradient (Fig. 4A), and none of the PKSs found were shared among the sampling years. Only one GCF of PKSI was shared between WB14 and WB15; three were shared between WB14 and WB17; and two GCFs were shared between WB15 and WB17. Conversely, NRPS showed a lower replacement rate across the temporal gradient when compared to PKSI, sharing 77 GCFs between WB15 and WB17, and 4 were shared among all samples, suggesting that PKSI presents higher genetic diversity in the Whalers Bay biofilm microbial community than NRPS. The high replacement rate across the temporal gradient may be explained by the taxonomic differential distribution contributing to the PKSI and NRPS diversity (Fig. 4B). For instance, only Burkholderiaceae and Rhodobacteriaceae contributed to PKSI and NRPS diversity in all sampled years. Five taxonomic families were exclusively found in WB14; nine were found only in WB15; and two were found only in WB17 (Fig. 4B).

The results revealed intriguing insights into the dynamics of PKSI and NRPS abundance and genetic diversity in the Whalers Bay biofilm microbial community across multiple sampling years. Particularly, PKSI exhibited higher abundances in WB14, while NRPS displayed consistent abundance levels across the years. The evaluation of genetic diversity using BGCs grouped into GCFs unveiled a remarkable pattern of high replacement rates for both PKSI and NRPS, with no shared PKSs observed between sampling years. These findings suggest the intricate interplay between temporal dynamics, taxonomic distribution, and the genetic diversity of PKSI and NRPS in the Whalers Bay biofilm microbial community. Furthermore, the exclusive contributions of specific taxonomic families in different sampling years further highlight the dynamic nature of these biosynthetic pathways within this ecosystem and align with the concept that the diversity and distribution of BGCs in the environment are contingent upon the phylogenetic diversity within the microbial community (73).

### BGCs distribution across the spatial gradient in Whalers Bay biofilm microbial community

**Fig 5:**
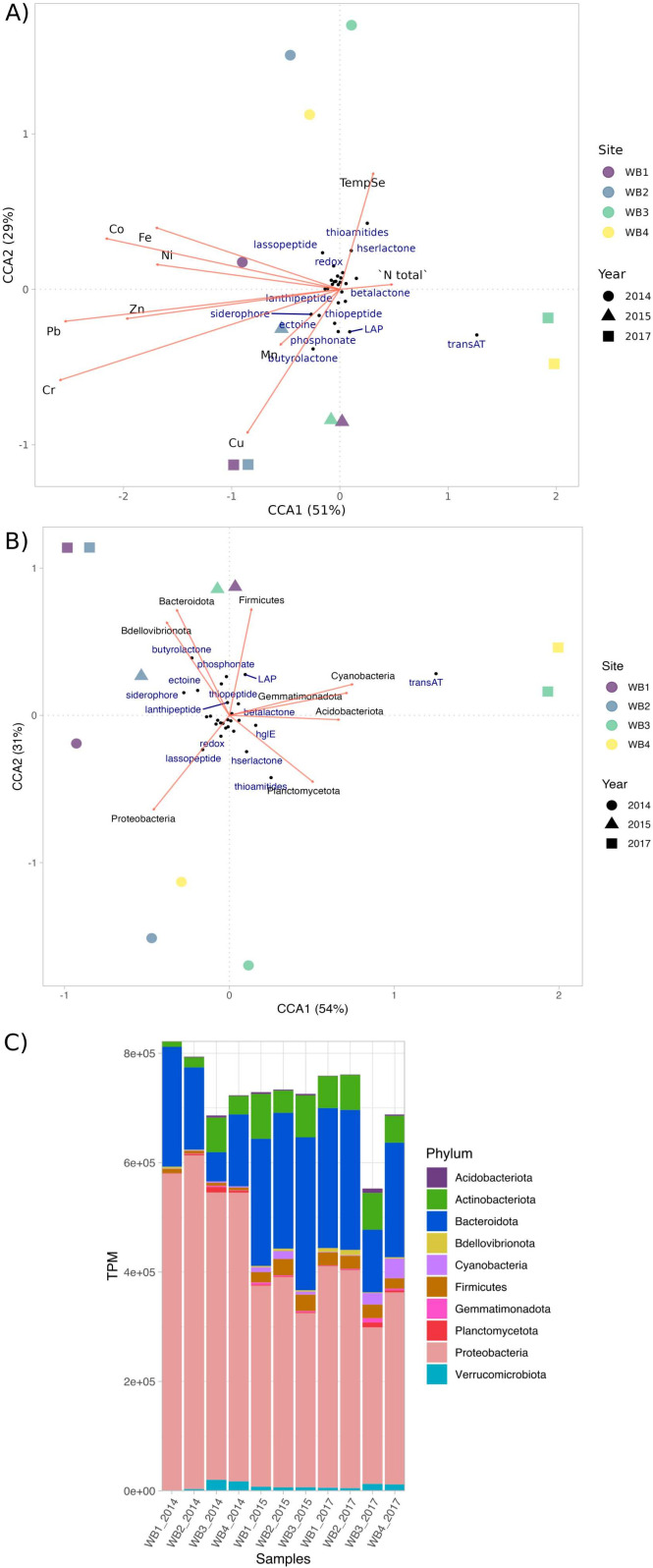
Biosynthetic gene clusters (BGCs) and taxonomic distribution in the Whalers Bay Biofilm Microbial Community. (**A**) Canonical correlation analysis illustrates the distribution of BGC abundances in response to environmental variables. The sites along the transect are denoted as WB1 (near the glacier) through WB4 (near the coast), with different shapes indicating sampling years. (**B**) Canonical correlation analysis showcases the distribution of BGC abundances relative to taxonomic groups. (**C**) Taxonomic diversity assessed across both the years (temporal gradient) and the transect sampling (spatial gradient). The transcripts per million (TPM) normalization method was applied to compare abundances among samples.

The BGC distribution along the spatial gradient (from near the glacier toward the coast) showed statistical differences (ANOVA, *P* value ≤0.05) for some predicted products, and these results varied for each year (Fig. S5 to S7). For instance, in WB14 and WB15, BGC encoding aryl polyene did not exhibit statistical differences along the spatial gradient (Fig. S5 and S6). In contrast, in WB17, aryl polyene showed higher abundance when closer to the coast (Fig. S7). On the contrary, siderophores showed higher abundances closer to the glacier in all years. Thus, a CCA was carried out to investigate the influence of the environmental variables in the BGC spatial distribution. The results suggest a metal-richer region closer to the glacier (Fig. 5A), but none of the predicted products could be strongly correlated to this feature. In addition, only phosphonate and butyrolactone seemed to respond to copper, and thioamides and hserlactone showed a positive correlation to temperature. Finally, TransAT-PKSs appeared to be correlated to the coast’s proximity and negatively correlated to metal-rich regions. Indeed, many BGCs are only transcribed with specific triggers (74). Therefore, environmental conditions may not directly select the BGC in the environment but may trigger BGC expression instead. Therefore, transcriptome data could improve the CCA analysis to explain BGC abundance and distribution in response to environmental conditions.

Considering the compelling evidence suggesting that taxonomic diversity plays a pivotal role in shaping the diversity and distribution of BGCs in the environment (5, 73), we conducted an assessment of taxonomic diversity across the spatial gradient and then correlated it with BGC distribution using a CCA. Proteobacteria was the most abundant phylum in all samples and showed higher abundances closer to the glacier, and Lasso peptide was positively correlated to this phylum (Fig. 5B and C). Bacteroidota appeared as the second most abundant phylum and, together with Firmicutes and Bdellovibrionota, contributed to explaining the abundances of butyrolactone, phosphonate, thiopeptides, and linear azol(in)e-containing peptides. The CCA results confirmed the role of taxonomic diversity in driving BGC abundance and distribution in the environment. TransAT-PKSs, for instance, showed a pronounced association with coast proximity, positively correlating with Cyanobacteria, Gemmatimonadota, and Acidobacteriota. Planctomycetota exhibited elevated abundances nearer to the coast, significantly influencing hserlactone and thioamide distribution patterns (Fig. 5B and C). These findings contribute to the understanding of the complex factors governing BGC abundance and distribution in this environment.

### Conclusions

This study unveiled the abundance and distribution of BGCs in the Whalers Bay biofilm microbial community in Antarctica. Firstly, the terpene BGC class emerged as dominant, accounting for nearly one-third of all identified BGCs, thereby underscoring their pivotal role in adapting to harsh environmental conditions. Particularly noteworthy is the high genetic diversity observed in PKSI and NRPS classes, along with their turnover rates over time, indicating their potential as reservoirs for novel bioactive compounds. Furthermore, the significant presence of BGCs encoding for potential antibiotics and immunosuppressive molecules draws attention to the pharmaceutical relevance of this polyextreme ecosystem. Moreover, the utilization of computational approaches in identifying known NRPs serves as a critical bridge between genetic potential and functional metabolites, paving the way for the discovery of bioactive compounds with diverse therapeutic applications.

The bacterial taxonomic diversity of the Whalers Bay biofilm, primarily composed by Proteobacteria, Bacteroidota, and Actinobacteriota, mirrors analogous observations in other Antarctic regions. This shared pattern highlights the robustness and adaptability of these microbial groups in the face of the harsh environmental conditions of the area. Nonetheless, distinctive associations between phyla and BGC abundance in Whalers Bay, in comparison to analogous environments, highlight the influence of local environmental conditions in structuring microbial communities and their biosynthetic potential. Finally, the multivariate analysis provided insights into the role of environmental variables in shaping BGC distribution patterns, offering a deeper understanding of the ecological drivers influencing specialized metabolite abundances. Overall, this study provided comprehensive information about the untapped biosynthetic potential of Antarctic microorganisms, shedding light on the intricate interplay between environmental gradients, taxonomic diversity, and specialized metabolite production. These findings also contribute to our broader understanding of microbial ecology in extreme environments, emphasizing the need for conservation of such invaluable ecosystems.

## SUPPLEMENTAL MATERIAL

Figure S1 spectrum.00244-24-s0001.pdf Site and sampling.

Figure S2 spectrum.00244-24-s0002.pdf Comparison of Nonpareil Curves for Whalers Bay Metagenomes in Deception Island, Antarctica.

Figure S3 spectrum.00244-24-s0003.pdf Sequence Similarity Network of Biosynthetic Gene Clusters (BGCs).

Figure S4 spectrum.00244-24-s0004.pdf Temporal Gradient: Abundance of predicted products over sample years.

Figure S5 spectrum.00244-24-s0005.pdf Spatial Gradient: Abundance of predicted products along the transect sampling.

Figure S6 spectrum.00244-24-s0006.pdf Spatial Gradient: Abundance of predicted products along the transect sampling.

Figure S7 spectrum.00244-24-s0007.pdf Spatial Gradient: Abundance of predicted products along the transect sampling.

Figure legends spectrum.00244-24-s0008.docx Full description of the figures and supplemental materials.

Supplemental tables spectrum.00244-24-s0009.xlsx Tables S1-S3.

ASM does not own the copyrights to Supplemental Material that may be linked to, or accessed through, an article. The authors have granted ASM a non-exclusive, world-wide license to publish the Supplemental Material files. Please contact the corresponding author directly for reuse.
