# Supplementary figures and images for: Unlocking the biosynthetic potential and taxonomy of the Antarctic microbiome along temporal and spatial gradients
Source: Microbiol Spectr. 2024 May 15;12(6):e00244-24. doi: 10.1128/spectrum.00244-24 (PMC11237469; doi:10.1128/spectrum.00244-24)

**B**

Sites 1 and 2

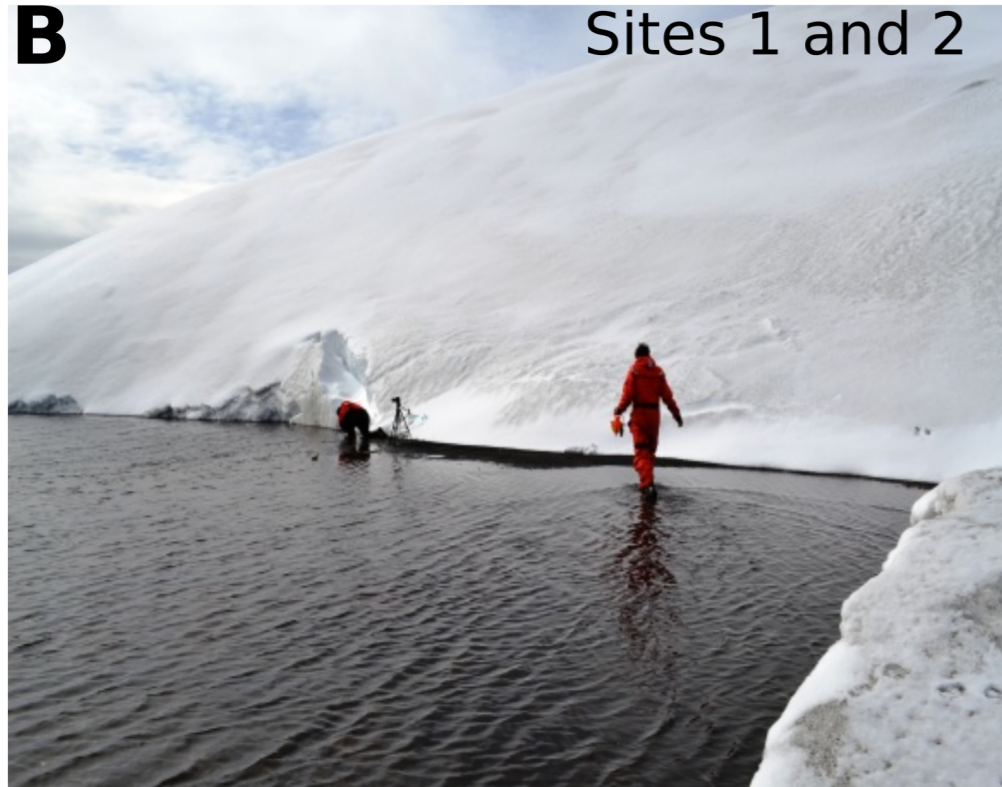

Site 3

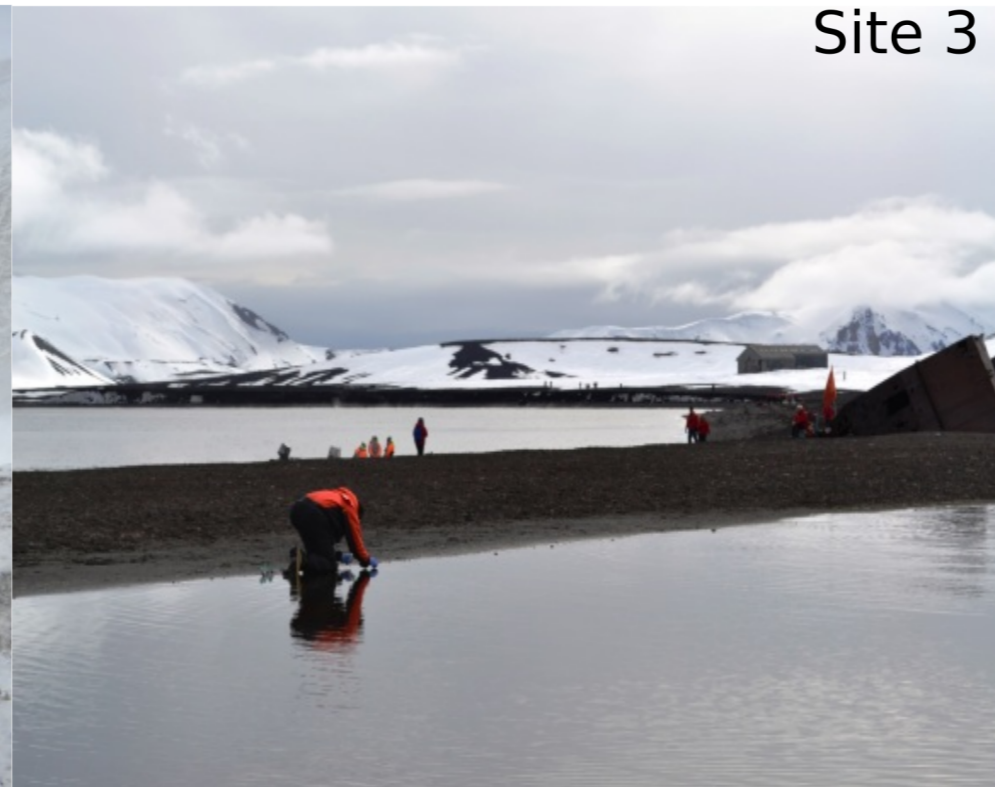

Site 4

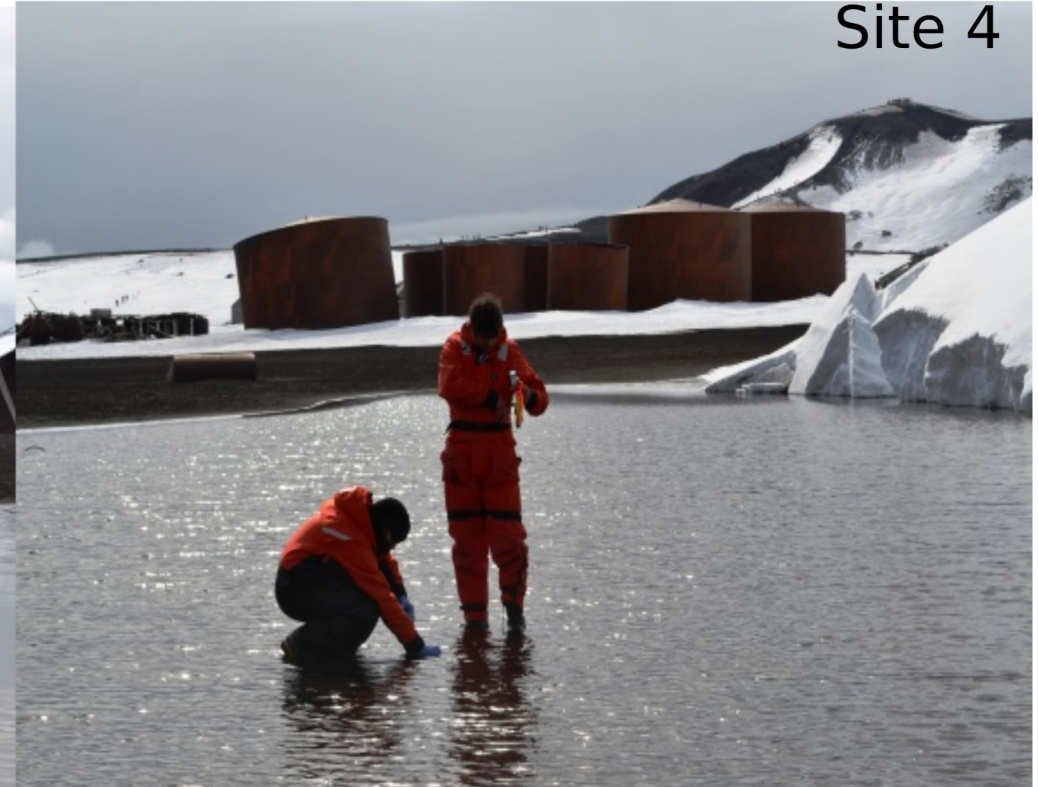**A**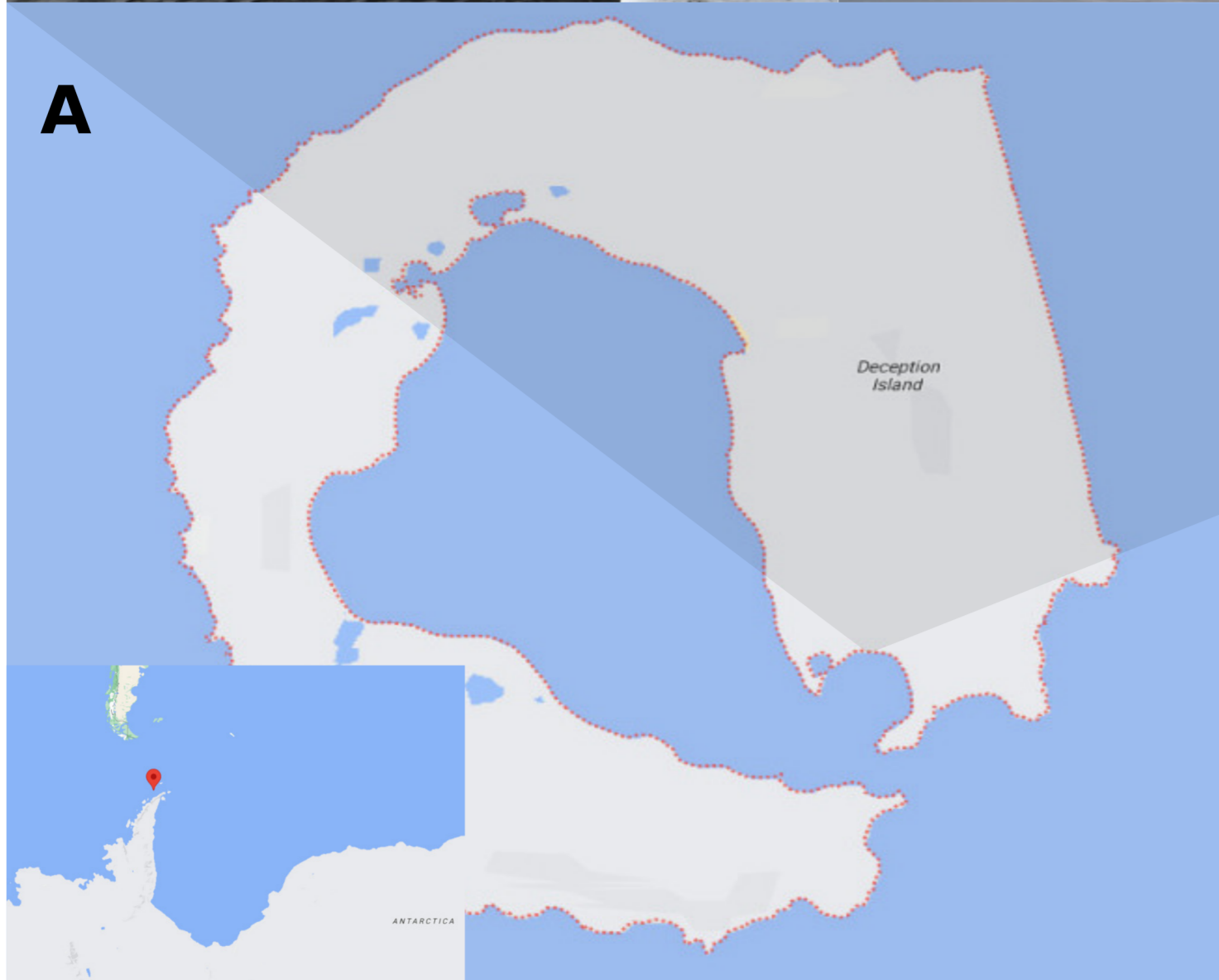**C**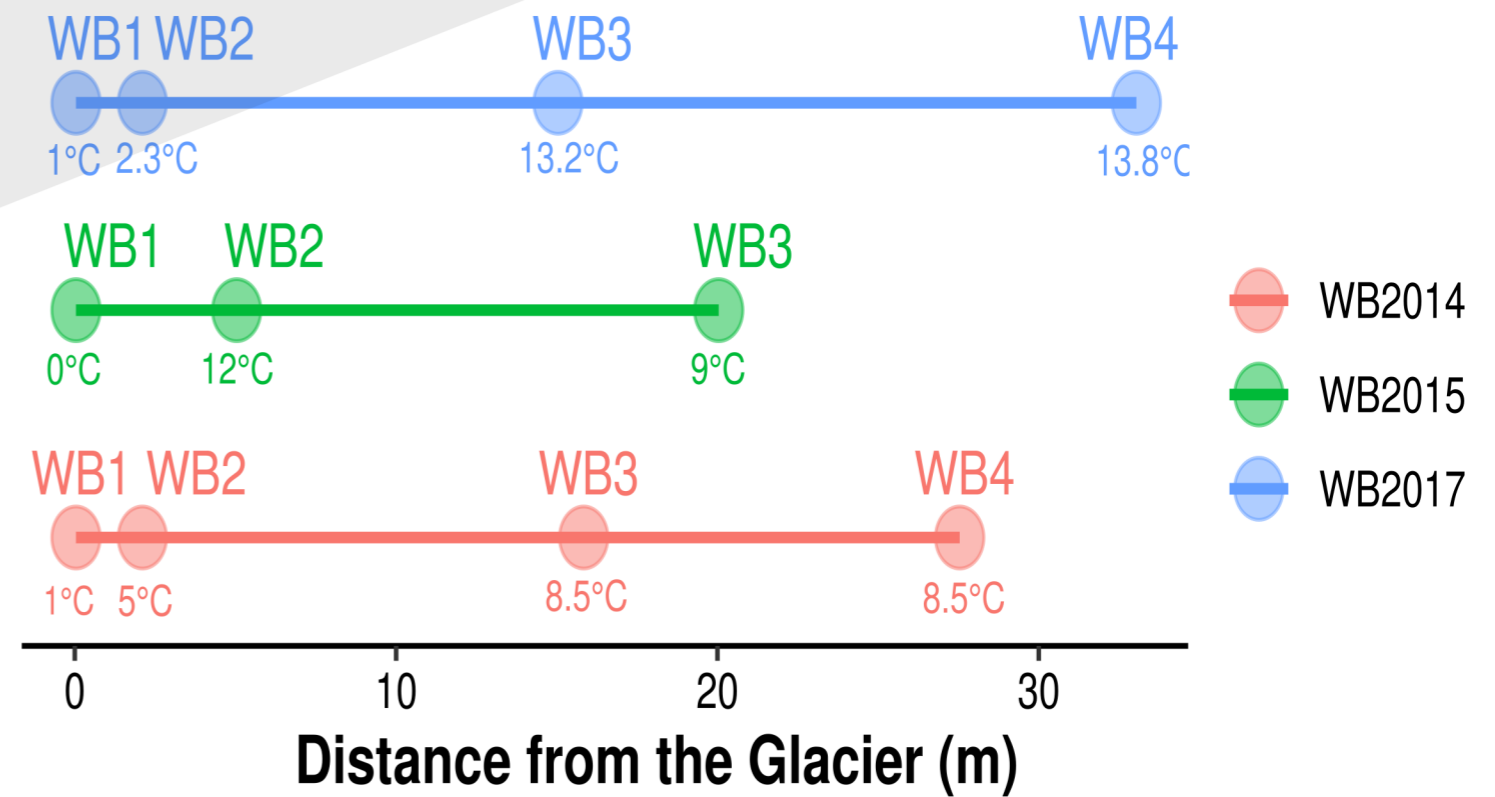

Supplement: Figure S1 — Site and sampling. [file spectrum.00244-24-s0001.pdf]

## Nonpareil Curves

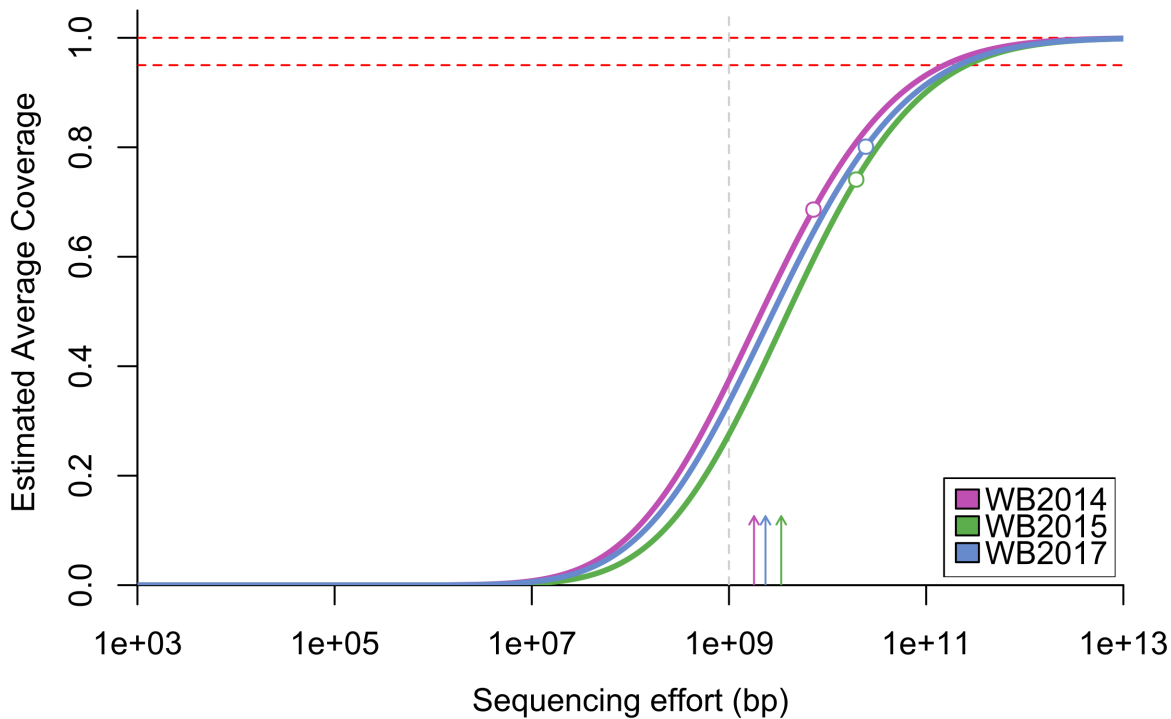

Supplement: Figure S2 — Comparison of Nonpareil Curves for Whalers Bay Metagenomes in Deception Island, Antarctica. [file spectrum.00244-24-s0002.pdf]

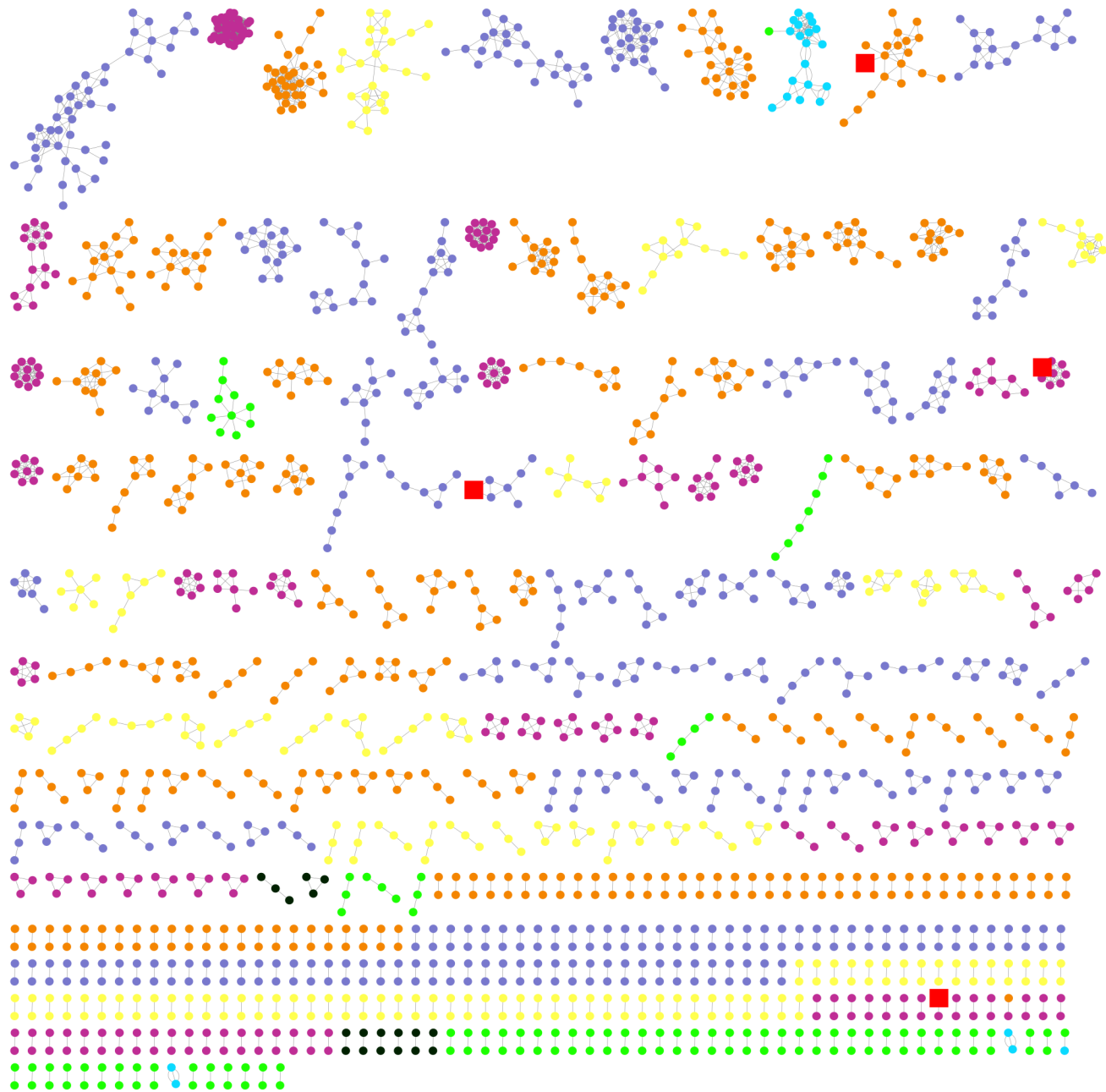

BGC class

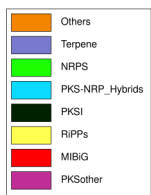

Supplement: Figure S3 — Sequence Similarity Network of Biosynthetic Gene Clusters (BGCs). [file spectrum.00244-24-s0003.pdf]

Sample 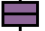 WB2014 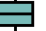 WB2015 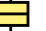 WB2017

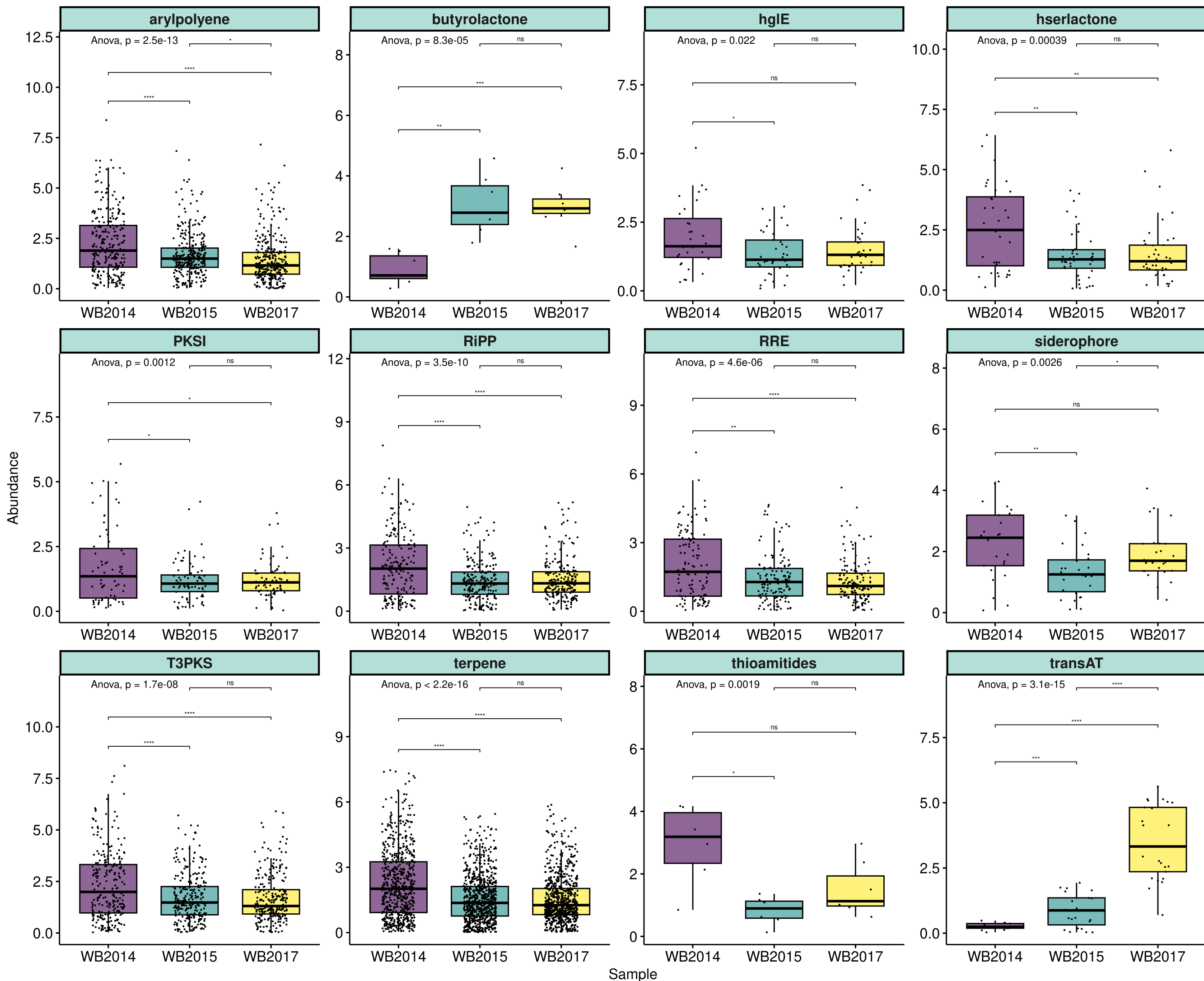

Supplement: Figure S4 — Temporal Gradient: Abundance of predicted products over sample years. [file spectrum.00244-24-s0004.pdf]

# Abundance of predicted products along the transecting sampling in 2014

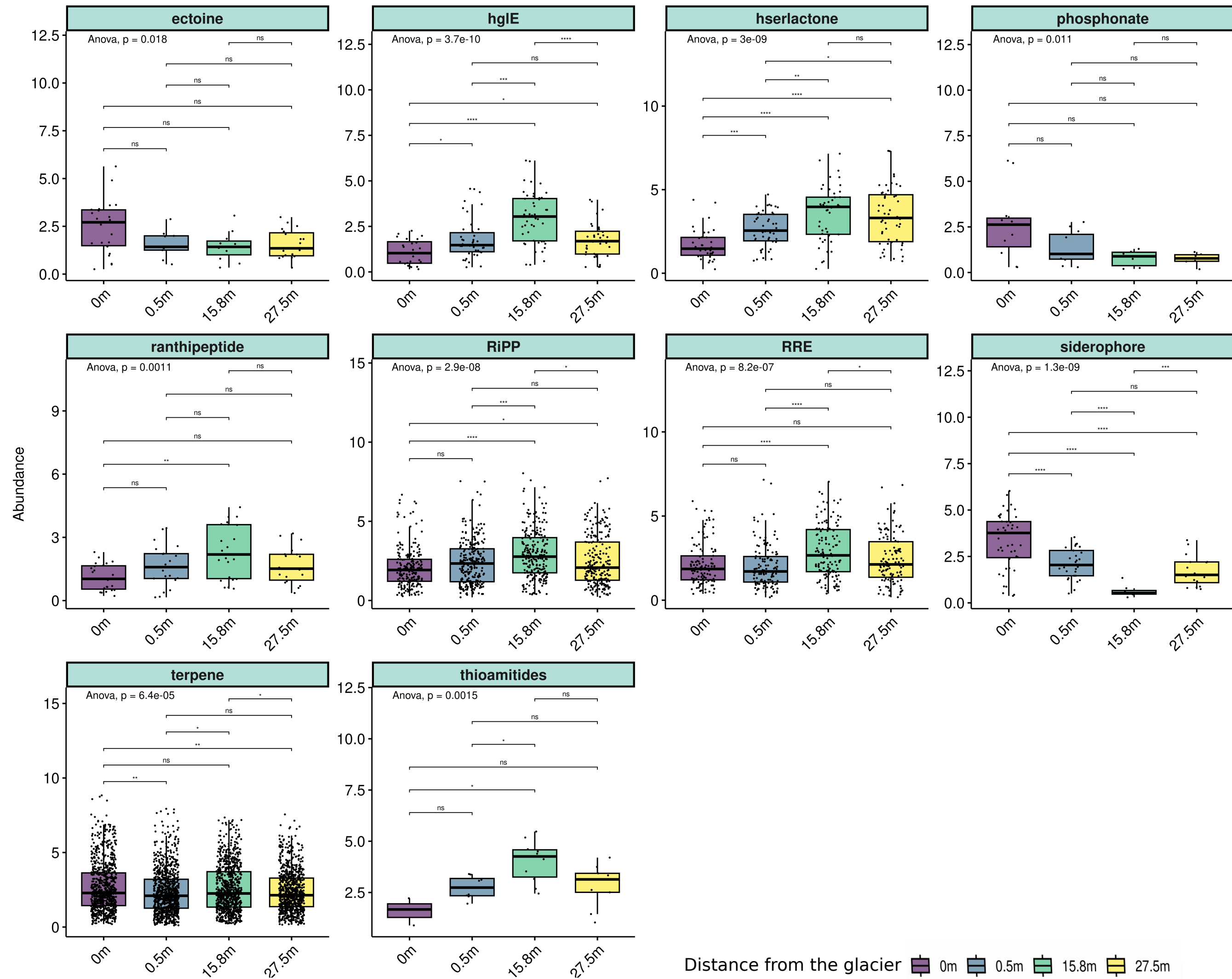

Supplement: Figure S5 — Spatial Gradient: Abundance of predicted products along the transect sampling. [file spectrum.00244-24-s0005.pdf]

Abundance of predicted products along the transecting sampling in 2015

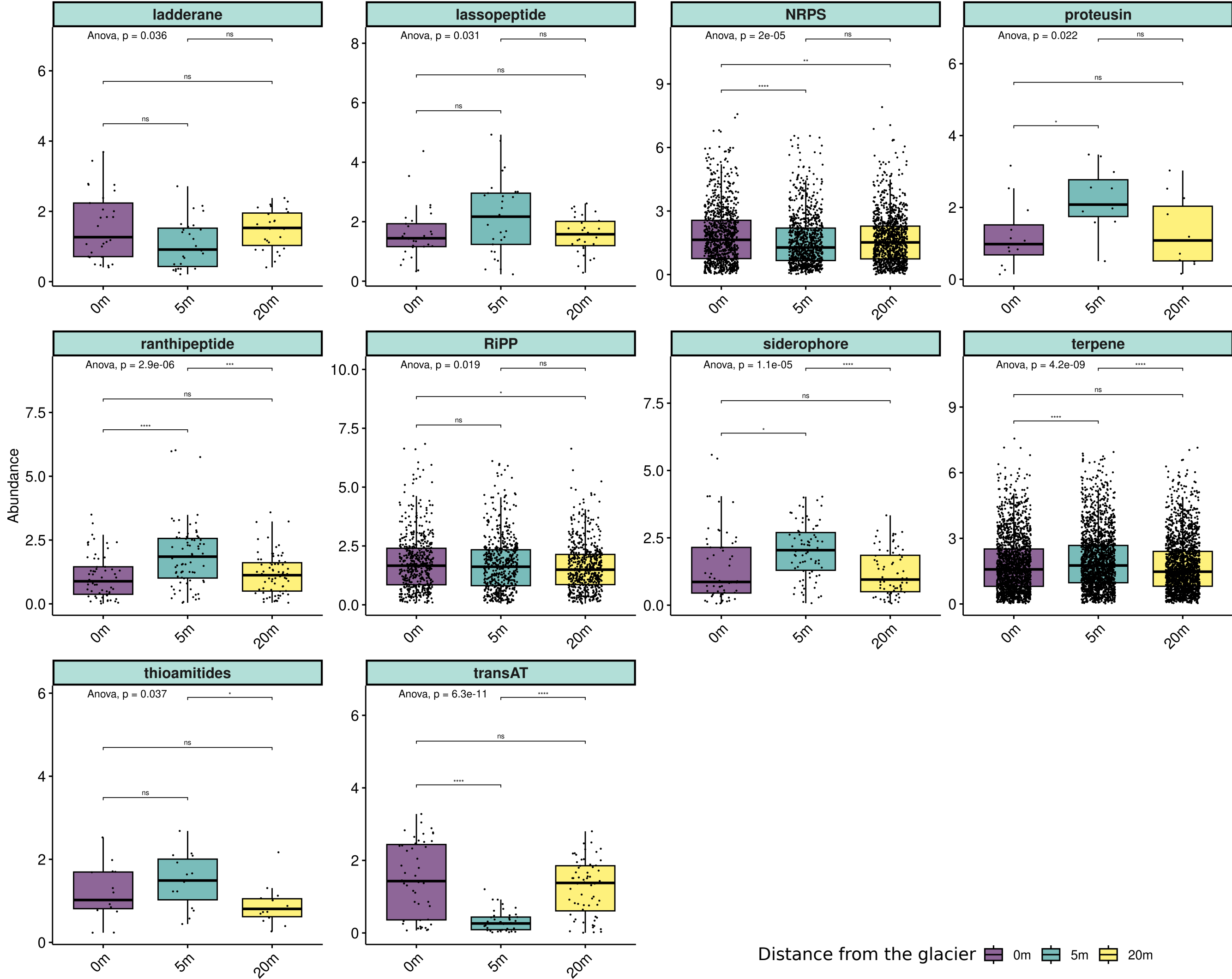

Supplement: Figure S6 — Spatial Gradient: Abundance of predicted products along the transect sampling. [file spectrum.00244-24-s0006.pdf]

Abundance of predicted products along the transecting sampling in 2017

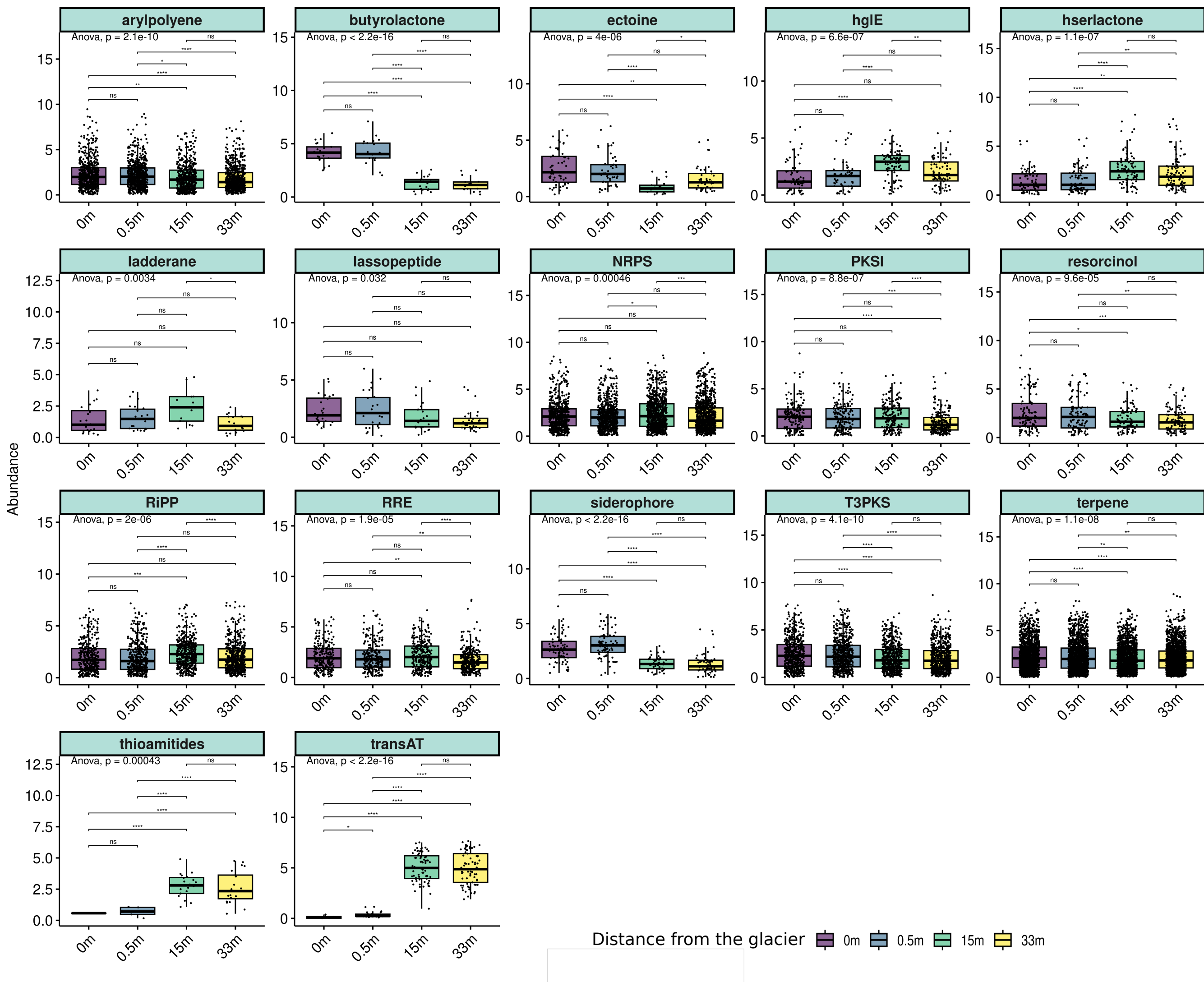

Supplement: Figure S7 — Spatial Gradient: Abundance of predicted products along the transect sampling. [file spectrum.00244-24-s0007.pdf]
