# Supplementary material for: Unlocking the biosynthetic potential and taxonomy of the Antarctic microbiome along temporal and spatial gradients
Source: Microbiol Spectr. 2024 May 15;12(6):e00244-24. doi: 10.1128/spectrum.00244-24 (PMC11237469; doi:10.1128/spectrum.00244-24)
Supplement: Figure legends — Full description of the figures and supplemental materials. [file spectrum.00244-24-s0008.docx]

List of legends

Fig. 1 – Biosynthetic Gene Cluster Classes and Predicted Products Identified in the Whalers Bay Biofilm Microbial Community. A) Abundances of Biosynthetic Gene Cluster (BGC) classes. B) Predicted products encoded within each BGC class. Circle sizes correspond to the predicted product abundances.

Fig. 2 – Bacterial Diversity in the Whalers Bay Biofilm of Deception Island, Antarctica, and Biosynthetic Gene Cluster (BGC) Counts by Phylum. A) Relative abundances of the top ten bacterial phyla identified in Whalers Bay. B) Distribution and counts of BGCs within the top 15 phyla harboring distinct BGC classes.

Fig. 3 – Chemical structures of known Non-Ribosomal Peptides connected to BGCs identified in Whalers Bay, Deception Island - Antarctica.

Fig. 4 – PKS I and NRPS Intersections and Distribution. A) The Upset plot illustrates the overlap of PKS I and NRPS clusters observed over the sampled years. The top bar plot in each panel indicates the intersection size (number of clusters) in the combined sample sets, as reflected in the matrix below. Shared clusters among samples are denoted by dots connected with straight lines. B) Taxonomic family distribution within samples harboring PKS I and NRPS clusters.

Fig. 5 – Biosynthetic Gene Clusters (BGC) and Taxonomic Distribution in the Whalers Bay Biofilm Microbial Community. A) Canonical correlation analysis illustrates the distribution of BGC abundances in response to environmental variables. The sites along the transect are denoted as WB1 (near the glacier) through WB4 (near the coast), with different shapes indicating sampling years. B) Canonical correlation analysis showcases the distribution of BGC abundances relative to taxonomic groups. C) Taxonomic diversity assessed across both the years (temporal gradient) and the transect sampling (spatial gradient).

Supplementary figures

Supplementary Fig. 1 – Site and sampling. A – Deception Island, Antactica Peninsula, Whalers Bay is indicated. B – Samples sites. C – Sampling line, starting from near to the glacier toward to coast as represented by the circles. In 2014 and 2017 four samples were collected along the transect and in 2015 three samples were collected along the transect

Supplementary Fig. 2 – Comparison of Nonpareil Curves for Whalers Bay Metagenomes in Deception Island, Antarctica. The plot exhibits the fitted models of the Nonpareil curves. Horizontal dashed lines signify the 95 – 100% interval, representing near-complete coverage. Empty circles denote dataset size and estimated average coverage. Subsequent lines project the fitted model. Arrows indicate required sequencing effort for near-complete coverage attainment.

Supplementary Fig. 3 – Sequence Similarity Network of Biosynthetic Gene Clusters (BGCs) identified using antiSMASH and clustered with BiG-SCAPE at a similarity cutoff of ≥ 70% identity. This network delineates gene cluster families (GCFs), with connected lines denoting shared BGCs. Each node corresponds to a specific BGC class, distinguished by color. Red squares indicate GCFs with references in the MiBIG database.

Supplementary Fig. 4 – Temporal Gradient: Abundance of predicted products over sample years. Displayed are products with statistically significant differences (ANOVA, p-value ≤ 0.05)

Supplementary Fig. 5 – Spatial Gradient: Abundance of predicted products along the transect sampling. Sites are labeled as WB1 (0m from the glacier), WB2 (0.5m from the glacier), WB3 (15.8m from the glacier), and WB4 (27.5m from the glacier). Illustrated are predicted products with statistically significant differences (ANOVA, p-value ≤ 0.05).

Supplementary Fig. 6 – Spatial Gradient: Abundance of predicted products along the transect sampling. Sites are labeled as WB1 (0m from the glacier), WB2 (5m from the glacier), and WB3 (20m from the glacier). Illustrated are predicted products with statistically significant differences (ANOVA, p-value ≤ 0.05).

Supplementary Fig. 7 – Spatial Gradient: Abundance of predicted products along the transect sampling. Sites are labeled as WB1 (0m from the glacier), WB2 (0.5m from the glacier), WB3 (15m from the glacier), and WB4 (33m from the glacier). Illustrated are predicted products with statistically significant differences (ANOVA, p-value ≤ 0.05).
